# Non-antibacterial tetracycline formulations: clinical applications in dentistry and medicine

**DOI:** 10.3402/jom.v4i0.19227

**Published:** 2012-10-12

**Authors:** Ying Gu, Clay Walker, Maria E. Ryan, Jeffrey B. Payne, Lorne M. Golub

**Affiliations:** 1 Department of General Dentistry, School of Dental Medicine, Stony Brook Medicine, Stony Brook University, Stony Brook, NY, USA; 2 Department of Oral Biology, School of Dental Medicine, University of Florida at Gainesville, Gainesville, FL, USA; 3 Department of Oral Biology and Pathology, School of Dental Medicine, Stony Brook Medicine, Stony Brook University, Stony Brook, NY, USA; 4 Department of Surgical Specialties, College of Dentistry, University of Nebraska Medical Center, Lincoln, NE, USA

**Keywords:** tetracyclines, host-modulation, matrix metalloproteinase inhibitors, clinical applications

## Abstract

In 1983, it was first reported that tetracyclines (TCs) can modulate the host response, including (but not limited to) inhibition of pathologic matrix metalloproteinase (MMP) activity, and by mechanisms unrelated to the antibacterial properties of these drugs. Soon thereafter, strategies were developed to generate non-antibacterial formulations (subantimicrobial-dose doxycycline; SDD) and compositions (chemically modified tetracyclines; CMTs) of TCs as host-modulating drugs to treat periodontal and other inflammatory diseases. This review focuses on the history and rationale for the development of: (a) SDD which led to two government-approved medications, one for periodontitis and the other for acne/rosacea and (b) CMTs, which led to the identification of the active site of the drugs responsible for MMP inhibition and to studies demonstrating evidence of efficacy of the most potent of these, CMT-3, as an anti-angiogenesis agent in patients with the cancer, Kaposi's sarcoma, and as a potential treatment for a fatal lung disease (acute respiratory distress syndrome; ARDS). In addition, this review discusses a number of clinical studies, some up to 2 years’ duration, demonstrating evidence of safety and efficacy of SDD formulations in humans with oral inflammatory diseases (periodontitis, pemphigoid) as well as medical diseases, including rheumatoid arthritis, post-menopausal osteopenia, type II diabetes, cardiovascular diseases, and a rare and fatal lung disease, lymphangioleiomyomatosis.

Four to five decades ago, a new concept was introduced regarding the pathogenesis of periodontitis, namely, that the host response, and not proteinases and other virulence factors produced by the microbial bio-film (then called the dental plaque), is primarily responsible for the connective tissue breakdown, including alveolar bone loss, which defines this most common of all chronic inflammatory diseases. The key paradigm-changing studies at that time which highlighted the host response included those by:

- Lehner and colleagues (1) in the United Kingdom who began to elucidate the role of the immune response in periodontal breakdown followed, a decade later, by Taubman and colleagues (2), including those studies using germ-free rats which supported the important role of T-cells and B-cells in periodontal bone loss.
- Fullmer and Gibson (3) at the National Institutes of Health/National Institute of Dental Research who, using the collagen-degrading tissue culture system of Gross and LaPiere (4), demonstrated for the first-time the pivotal role of host-derived (i.e. human tissue) collagen-destructive enzymes (the collagenases and other matrix metalloproteinases or MMPs) in periodontal tissue destruction, including bone resorption.

Since then, the widely accepted sequence of biological events in the pathogenesis of chronic periodontitis has been the one outlined previously by Ryan and Golub (5) as well as others (see Fig. 1). This overview, which addresses the contribution of the microbial biofilm (particularly Gram-negative anaerobic microorganisms, such as *P gingivalis*) as the *initiator* of the inflammatory response, but which highlights the role of pathologically excessive MMPs as well as elevated levels of inflammatory mediators (cytokines, prostanoids, reactive oxygen species, inducible nitric oxide synthase), led to the search for host-modulating drugs for treating periodontitis. These pharmaceuticals included the non-steroidal anti-inflammatory drugs (NSAIDs) (6) and, more recently, resolvins (7) to, respectively, suppress and prevent the prolongation of inflammation, as well as the bisphosphonates to inhibit osteoclast-mediated bone resorption (8). However, the only host-modulating drug that has been approved by the United States Food and Drug Administration (FDA) and other national regulatory agencies in Canada and Europe as adjunctive treatment for the management of chronic periodontitis is a novel ‘low-dose’ formulation (20 mg b.i.d., compared to ‘regular or antibiotic-dose’ 100 mg q.d. or b.i.d.) of a tetracycline (TC), doxycycline (9, 10). This non-antibiotic formulation of doxycycline, better known as subantimicrobial-dose doxycycline (SDD) or Periostat^®^ (10, 11), was designed to: (a) suppress host-derived MMPs in the periodontal lesion thereby inhibiting the pathologic degradation of various collagens, including types I, III, and IV, while preserving other constituents of the periodontal tissues (fibronectin, proteoglycan ground substance, elastin and basement membranes) and inhibiting bone resorption, and (b) prevent complications of ‘regular-dose’ TC (e.g. doxycycline) administration, such as gastrointestinal disturbance, increased photosensitivity and the emergence of antibiotic-resistant microorganisms (see next section).

**Fig.1:**
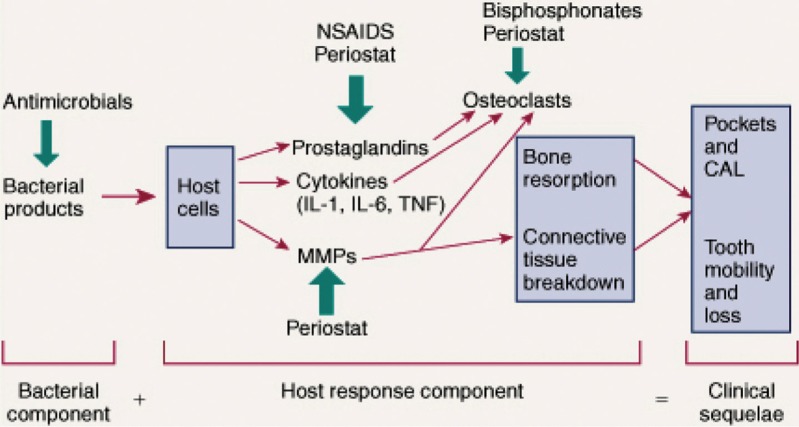
Schematic illustration of the current view of the pathogenesis of periodontitis and potential adjunctive therapeutic approaches. CAL, clinical attachment loss. This figure was published in Carranza's Clinical Periodontology, 11th ed., Newman MG, Takei HH, Klokkevold PR, Carranza FA. in Chapter 48, Host Modulation, Ryan ME, Gu Y., Page 493. Copyright © 2006 by Saunders, an imprint of Elsevier Inc.

The rationale for developing a non-antimicrobial formulation of a TC (doxycycline was selected because it was a more potent inhibitor of MMPs than other TCs; exhibited superior pharmacokinetics which favoured improved patient compliance; was safer than minocycline (9–11)) arose out of early discoveries by Golub et al. (11–14) using the diabetic rat model. In brief, this group had determined that a number of complications of diabetes, including severe periodontal breakdown, were associated with abnormalities in collagen structure and turnover. They were the first to discover that experimentally inducing type I diabetes (and later type II) increased the production and activity of mammalian collagenase in *any* tissue, although the tissues they were studying were the gingiva, providing one explanation for the unusually aggressive periodontal breakdown often associated with this hyperglycemic condition (12–15). In an effort to determine whether a diabetes-induced alteration in the oral microflora played a role in the enhanced host-derived collagenase activity in gingival tissues, Golub and colleagues suppressed the bacteria by treating the diabetic rats with a TC, minocycline, which reduced this collagen-destructive enzyme activity (13). However, when these investigators repeated this experiment, except with germ-free rather than conventional rats, remarkably they found: (a) that diabetes still increased collagenase production/activity in the gingival tissues indicating that an altered host response (e.g. the formation of advanced glycation end-products or AGEs), even in the absence of an oral microflora, can increase mediators of periodontal breakdown and, (b) that treating the germ-free diabetics with a TC *still* reduced the pathologically excessive mammalian collagenase/collagen-destructive enzyme activity in the gingiva (13). As a result of these seminal studies plus later experiments demonstrating that TCs (but not other antibiotics) can also directly inhibit various MMPs *in vitro* and reduce these neutral proteinases in other tissues (e.g. skin) and a variety of cell types (e.g. acute and chronic inflammatory cells, bone and cartilage cells, cancer cells), these investigators concluded that only TCs, and not other antibiotics, can inhibit collagenases and other host-derived MMPs, and by mechanisms *independent* of the antibacterial properties of these drugs (11, 13, 14). They subsequently identified the multiple (pleiotropic) mechanisms by which TCs inhibit MMPs and suppress connective tissue and bone destruction, and identified the active site, a metal ion (calcium and zinc)-binding β-diketone moiety at carbon-11 and carbon-12 of the TC molecule (11, 14, 16).

The group's *first strategy*, described above, to develop non-antibacterial formulations of TCs for clinical use, SDD, has since demonstrated safety and efficacy in phase III clinical trials and has been approved by the US FDA: (a) as the first-ever systemically administered drug for the management of periodontal disease (adjunctive to scaling and root planing, SRP), that is, Periostat^®^, now generic; and (b) as a NOVEL sustained-release SDD formulation called Oracea^®^, which has also demonstrated efficacy in randomised clinical trials as an adjunct to SRP (17), and which is now approved (FDA and in Europe) as a new systemic treatment for the chronic inflammatory skin disease, rosacea; moreover, Oracea^®^ has also shown efficacy in the treatment of acne in adults (18–20). As discussed in later sections of this review, SDD formulations have recently demonstrated evidence of safety and efficacy in humans with a variety of other diseases and conditions as well, including pemphigoid, rheumatoid arthritis (RA), post-menopausal (PM) osteopenia, type II diabetes, cardiovascular diseases and a rare and fatal lung disease, lymphangioleiomyomatosis (see below).

As a result of the discovery of the active site for at least many of the pleiotropic mechanisms by which TCs inhibit collagenolysis, the *second strategy* of the group was to chemically modify the TC molecule to eliminate its antibiotic activity (by removal of the dimethylamino group at carbon-4 of the ‘A’ ring (16)) but to retain its zinc-binding β-diketone site (see above) to preserve, or even enhance, its anti-collagenase activity (11, 14, 16). As a result, an initial series of chemically modified TCs, CMTs 1–10 (14), were developed and one of the most potent MMP inhibitors of this series, CMT-3 (6-demethyl 6-deoxy 4-de-dimethylamino TC; also known as COL-3), has been tested in phase II clinical trials by Dezube et al. (21), through a cooperative research and development agreement (CRADA) with the National Cancer Institute (NCI), on patients with Kaposi's sarcoma, and has shown efficacy as an anti-angiogenesis agent (21, 22). The same compound, CMT-3, significantly reduced mortality in a Yorkshire pig model and other animal models of a 40% fatal lung disease, acute respiratory distress syndrome (ARDS) and counteracts septic shock (23–26). However, this potent non-antimicrobial TC compound does produce clinically significant photosensitivity in a dose–response manner in human subjects (21, 22) resulting in a search for new molecules, with the same active site as the TCs but with a different phenolic superstructure, and these (the ‘PEZBINs’, polyenolic Zn^2 +^ binding compounds) are just beginning to be developed (23, 27).

## The development of novel tetracycline formulations: evidence in support of their lack of antibacterial activity

The TC family, which includes doxycycline, consists of broad spectrum bacteriostatic antibiotics that act by inhibiting bacterial protein synthesis. To be effective, such compounds must enter the bacterial cell in sufficient concentrations to bind to the ribosomal acceptor site on the 30S ribosomal subunit of the mRNA translation complex. There the drug prevents the incorporation of amino-acyl tRNA to the growing peptide chain. When present in sufficient concentrations, tetracyclines (TCs) block protein synthesis. Mechanisms of antibiotic resistance are primarily a function of ribosomal protection and non-specific efflux pumps. Ribosomal protection confers broad antibiotic resistance and involves the synthesis of a protein that either prevents the binding of the drug to the ribosomal site or results in dislodging the bound TC. The efflux pump is normally repressed, but when the repressor protein is removed by the recognition of a foreign substance, the pump is activated and ‘pumps’ the compound out of the cell before it can reach a critical antibacterially effective concentration. The efflux pump is especially effective against TC, to a lesser extent against doxycycline, and even less so against minocycline, the most lipophilic of the three antibiotics (28).

For TCs to exert an antimicrobial effect (and of relevance to any antibiotic), sufficient concentrations of the drug must reach the bacteria. The introduction of doxycycline in a sub-antimicrobial formulation as a therapeutic adjunct raised the question in some minds that even a sub-antimicrobial dose of a drug could lead to resistance. The FDA required that this possibility be thoroughly investigated in double-blinded clinical trials to determine the safety of long-term SDD prior to approval. The criteria for safety were as follows: no significant change in (a) susceptibility to doxycycline, (b) the proportion of periodontal pathogens present and (c) the emergence of opportunistic pathogens such as *Candida*, *Staphylococcus aureus*, or enterics.

The first major clinical safety study was conducted jointly at the University of Florida and West Virginia University. Seventy-six subjects with chronic periodontitis were enrolled in a double-blind study and randomly assigned to receive SDD, 20 mg b.i.d. or placebo. A split mouth design was used with each subject receiving subgingival SRP in two quadrants while the other two quadrants were left unscaled (non-SRP). Subgingival plaque samples were collected prior to treatment (baseline) and after 3, 6 and 9 months of treatment. Both control and experimental treatments resulted in statistically significant decreases in the proportions of spirochetes and motile rods (*p <* 0.05) and an increase in coccoid forms (*p <* 0.0001) relative to baseline. No between-treatment differences were detected between the SDD and placebo treatments in either the SRP or non-SRP design, with the exception of the small and large spirochetal groups. The spirochetal proportions present in the SDD group were significantly lower (*p <* 0.05) than the paired placebo group during the 9-month treatment and was preceded by a significant decrease (*p <* 0.01) in the proportion of microbiologic sample sites that bled on probing. No between-treatment differences were detected in any of the other microbial parameters (29). The proportions of the cultivable flora with resistance to doxycycline (4 µg/ml) and cross-resistance to non-TC antibiotics were also assessed. There were no statistically significant differences in the proportion of doxycycline-resistant isolates among treatment groups and no evidence of multi-antibiotic resistance or cross-resistance at any time point (30). Of the 658 susceptibility patterns evaluated, the MIC50/90 and mode MIC showed stable patterns, independent of treatment group (31).

It was concluded that the microbial differences observed in the proportions of spirochetes present were attributed to the anti-collagenase and anti-inflammatory properties of SDD modifying the environment in which the bacteria reside, and not to an antimicrobial effect, since there were no detectable changes in any of the other microbial parameters evaluated and long-term SDD did not lead to changes in antibiotic susceptibility (32).

A multi-centred clinical study by Preshaw et al. (17) examined the clinical and microbial effects of a novel sustained-release SDD formulation containing 40 mg doxycycline (SDD-40) taken once daily which produces low blood levels (*<*1 µg/ml) similar to 20 mg b.i.d. A total of 266 subjects with periodontitis were evaluated in a double-blind, placebo controlled multi-centre study over a 9-month period. Compliance with study medication was high (>92%) with no significant differences in adverse events between groups and no evidence of microbiologically significant changes or development of antibiotic resistance in the subgingival flora in either group. It was concluded that sustained-release of SDD-40 used as an adjunct to SRP resulted in significantly greater clinical benefits than SRP alone in the treatment of periodontitis and did not have an effect on the composition or the antibiotic-susceptibility of the subgingival flora. Similar results were reported in a review of the clinical, microbiologic and safety data for SDD administered as 20 mg b.i.d. (33).

Likewise, in a double-blind, placebo-controlled study that examined the intestinal flora in 69 periodontally diseased subjects that received a 9-month course of SDD, 20 mg b.i.d., there was no evidence that SDD exerted an effect on the composition or the susceptibilities of the faecal flora. In a smaller subset of these subjects, the vaginal flora was also evaluated and found to demonstrate no effect from the SDD treatment compared to placebo (34), unlike the development of opportunistic infections with yeast which is often seen during administration of higher antibiotic doses of doxycycline.

In a 6-month multi-centred, double-blind, randomised study that evaluated the effect of SDD in the treatment of moderate facial acne, the drug was found to be well tolerated and did not result in an increase in the number of the doxycycline-resistant skin bacteria (35).

In the longest SDD clinical study to date, a 2-year regimen of SDD was evaluated for its effects on osteopenic bone loss in PM estrogen-deficient women. The study was a two-centre, double-blind, placebo-controlled, randomised clinical trial in which each subject either took 20 mg b.i.d., SDD, or a placebo look-alike (b.i.d.) each day during the 2-year protocol. Subgingival samples were collected at baseline and at the end of the study. Statistical analyses were conducted between and within treatments for microbial differences in total colony-forming units (CFUs), periodontal and opportunistic pathogens, and changes in species and in susceptibilities of isolates to doxycycline and five other antibiotics. There was no significant evidence that changes in total anaerobic counts over the treatment period (*p =* 0.96) differed between treatment groups. Likewise, periodontal pathogens, opportunistic pathogens and normal flora did not differ descriptively between groups. Although there was a significant increase (*p <* 0.001) in the total CFUs recovered on the 4 µg/ml doxycycline plates at 24 months for SDD versus placebo, the percentage that was clinically resistant to doxycycline decreased over the 24-month period in both groups and did not differ between the treatment groups (SDD: 79 to 76%; placebo: 83 to 70%; *p =* 0.2). There were no significant differences (*p >* 0.28 for each) in the change in cross-resistance between the groups for doxycycline and the other five antibiotics (36).

The abundance of data now available clearly indicate that SDD, in either the 20 mg b.i.d. or the novel sustained-release 40 mg q.d. formulations, does not exert an antimicrobial effect on the human microflorae, regardless whether it is in the subgingival plaque, the colon, the vagina or the skin. This is not surprising when one considers that SDD, 20 mg b.i.d., only yields serum levels *<*1 µg/ml (37). When one also considers that the microflora in the studies cited above exists as bacterial biofilms, it should be understood that 1 µg of doxycycline/ml or less in serum (or even, at somewhat higher levels, in the gingival fluid) would not exert an antimicrobial effect on these biofilms. It has been demonstrated that antibiotic concentrations required to inhibit or kill bacteria within a mature biofilm are several orders of magnitude greater than that required to inhibit or kill the same bacterial strains when grown planktonically. For some antibiotics, concentrations of 1,000–2,000 µg (1–2 mg)/ml are required to demonstrate antibacterial effects on biofilms (38). A number of explanations have been advanced to account for the higher drug levels required to effect mature biofilms (39–42). These include (but are not limited to) the presence of the additional polysaccharide that surrounds the biofilm, the lower metabolic activity associated with mature biofilms, and the difficulty of antibiotics to thoroughly penetrate the biofilm matrix. Regardless of the mechanisms that may be involved, it is clear that biofilms are considerably more resistant to antibiotics than planktonic cultures. Thus, it is understandable (and clinical studies have demonstrated) that doxycycline levels *<*1 µg/ml have no direct antimicrobial effect on these biofilm microflorae, except for those non-antibacterial properties of SDD that reduce both inflammation and the degradation of protein constituents of the adjacent tissues which can alter the environment in the periodontal pocket in which the biofilm exists.

## A review of the dental and medical clinical applications of non-antibacterial tetracycline formulations

### Non-antibacterial tetracyclines for periodontal (and other oral) diseases

The history and rationale for the development of SDD has already been introduced earlier in this article and detailed in several review articles (5, 10, 11, 14) over the past two decades and, therefore, will only be summarised here. Immediately after the discovery, first described in 1983 (13), that TCs can inhibit host-derived collagenase (and later, other MMPs as well), and by mechanisms *independent* of the antibacterial property of these drugs, a strategy was developed to formulate a low-dose TC that could be administered long-term for the treatment and management of chronic periodontitis without antibiotic side-effects, such as photosensitivity, gastrointestinal disturbance and colonisation with antibiotic-resistant bacteria (11, 14, 20). Initially, a low-dose of minocycline was tested (43) but was soon replaced by low-dose doxycycline (10, 14). Moreover, these early studies demonstrated that a 2–3 week regimen of low-dose (now called subantimicrobial-dose) doxycycline, although it did reduce collagenase activity in the human periodontal pocket (the gingival crevicular fluid or GCF) and in the gingival tissue (44), did not produce a lasting effect; that is, upon cessation of drug therapy, the reduced collagenase activity rebounded back to the high levels seen in placebo-treated subjects (9). In contrast, longer term regimens of SDD (3–9 months duration) did produce a prolonged effect even after cessation of drug administration. As examples, in a phase III double-blind, placebo-controlled clinical trial on 190 subjects with chronic periodontitis, Caton et al. (45) found that a 9-month regimen of SDD adjunctive to SRP produced a therapeutic effect for at least 3 months after the cessation of drug therapy. In this important study, no rebound effect was observed in either pocket depth reductions or clinical attachment level gains; in fact, there appeared to be some continuing improvement in both of these clinical parameters, presumably because of the improved clinical status of the patients resulting from adjunctive SDD and also, perhaps, reflecting the persistence of doxycycline in the bone and soft tissues of the periodontium extending the duration of its efficacy. More recently, Emingil et al. (46) reported that a 3-month regimen of SDD adjunctive to non-surgical periodontal therapy produced a therapeutic benefit for at least a 12-month period.

Clearly, a major concern about the use of antibiotics as adjuncts to mechanical debridement procedures has been the development of antibiotic side-effects, and several studies have shown that short-term and long-term administration of regular-dose formulations of TCs (minocycline, doxycycline, TC itself) results in the emergence of antibiotic-resistant bacteria. Short-term (days to several weeks) treatment with minocycline or doxycycline can result in colonisation with resistant bacteria in the oral cavity and elsewhere (47–50) and in a study by Kornman and Karl (51), patients administered TC for a year (250mg 1 or 2/day) experienced a dramatic overgrowth of antibiotic-resistant fusobacterium in the subgingival plaque.

With this background in mind, several decades ago, experimental low-dose doxycycline formulations were prepared containing progressively lower amounts of the drug per capsule ranging from 100, 50, 40, 30, 20 and, finally, 10 mg. The goal was to achieve a pharmacokinetic profile in patients with ‘peak’ (Cmax) blood levels <1 µg/ml, in contrast to the Cmax of 2–5 µg/ml achieved by regular-dose doxycycline (50 mg b.i.d., or 100 mg q.d. or b.i.d.) (14, 52, 53). As a result of these early clinical trials, the dosage regimen of 20 mg b.i.d. was found to be safe and effective. More recently, a more advanced NOVEL sustained release formulation was designed (containing two types of doxycycline ‘beads’) which produced similar Cmax values of 0.5 µg/ml (single dose) and 0.6 µg/ml (steady-state), and which resulted in no adverse events significantly different from placebo-treated subjects, in extensive FDA-approved clinical trials.

As a result of several decades of extensive clinical trials, which have been described in many reviews (52, 54–57), SDD adjunctive to SRP has been shown to be effective in improving the standard parameters of periodontal disease severity, including probing depth, clinical attachment levels, bleeding-on-probing and radiologic assessment of alveolar bone loss; additional improvements seen in clinical trials include reduction in tooth loss, prevention of disease progression and decreased levels of diagnostic biomarkers of inflammation, collagenolytic activity and bone resorption in the periodontal pocket. Based on extensive, multi-institutional, double-blind, placebo-controlled clinical trials, SDD is the first ever MMP-inhibitor drug approved for *any* disease, and the first systemically administered drug approved by the US FDA, Canadian and European regulatory agencies as adjunctive host-modulating therapy for periodontitis. The evidence supporting the safety and efficacy of SDD has also been examined and accepted by the ADA's Council for Scientific Affairs, and has been confirmed by META statistical analyses in three separate publications by Reddy et al. (58), Preshaw et al. (59) and Sgolastra et al. (60) with the first of these reports (58) comprising part of a workshop by the American Academy of Periodontology on Contemporary Science and Clinical Periodontics, which designated the highest rating possible due to the strong and consistent evidence from properly randomised clinical trials.

More recently, SDD has been assessed for efficacy in more aggressive forms of periodontal disease. In brief, Novak et al. found that SDD (combined with repeated regimens of SRP) was 100% more effective clinically than this regimen of SRP plus placebo in patients with generalised aggressive periodontitis (61). The meta-analysis by Preshaw et al. (59) revealed a benefit in smokers treated with SDD as an adjunct to SRP, although the therapeutic effect in these patients, traditionally considered resistant to periodontal treatment, was not as dramatic as the effect of this treatment strategy in non-smokers. SDD treatment can also be combined with the local delivery of antibiotics into the periodontal pocket through sustained delivery systems. The two treatment approaches target different aspects of the pathogenic process: local delivery systems deliver antimicrobial concentrations of an antibacterial agent directly into the site of the pocket, whereas SDD is a systemic host response modulator. Thus, combining these two complementary treatment strategies is another example of how antibacterial therapy (SRP + local antibiotics) can be combined with host-modulation therapy (SDD) to maximise the clinical benefit for patients. Results from a 6-month, 180-patient clinical trial designed to evaluate the safety and efficacy of SDD combined with a locally applied antimicrobial (Atridox) and SRP versus SRP alone demonstrated that patients receiving the combination of treatments experienced more than a 2 mm improvement in mean attachment gains and probing depth reductions (p*<*0.0001) compared with SRP alone (62).

And, finally at a special clinic established at the University of Toronto which focuses on *Refractory Periodontitis*, Goldberg and Tenenbaum (63) have reported their preliminary studies indicating that a combination of SDD plus low-dose flurbiprofen, together with repeated bouts of non-surgical periodontal therapy, produces improvements in these traditionally non-responsive patients. The rationale for this ‘combination’ therapy originated from: (a) earlier studies on an animal model of arthritis which demonstrated therapeutic synergy of a non-antibiotic TC as an MMP-inhibitor drug combined with an NSAID (see section on Arthritis) and (b) a more recent clinical study demonstrating that a combination of SDD plus low-dose flurbiprofen resulted in the synergistic reduction of MMP (collagenase and gelatinase) and elastase activity in gingival tissues surgically excised for therapeutic purposes (44).

Regarding other oral inflammatory diseases, a pilot study carried out by Cohen et al. (64) indicated that patients with *benign mucous membrane pemphigoid*, who were treated with a 3-month regimen of SDD, showed a reduction in the blisters and ulcers in the oral mucosa which characterise this disease (also see section on Dermatologic diseases). The therapeutic rationale for this clinical strategy, which has also been anecdotally described by several oral pathologists in the USA (personal communication), may be linked to a recently described mechanism (65) involving the autoimmune induction of MMP-9 in the oral mucosa which, ultimately, contributes to the disruption of the hemidesmosomal protein, type XVII collagen, and blister formation, which could be prevented by the MMP-inhibitor, SDD.

### Dermatologic diseases

The medical discipline in which SDD currently has a very high and expanding level of clinical application is dermatology. As a reflection of this interest, an extensive review of this utility was recently published by Monk et al. (20) and, therefore, the abbreviated section below will only highlight some of the issues raised in that article. One of these issues is the recent development of a *modified* version of the original non-antibacterial formulation of doxycycline, Periostat (20 mg b.i.d.). This earlier SDD formulation was the first systemic medication approved by governmental regulatory agencies (USA, Canada and Europe) for the treatment (adjunctive to mechanical debridement) of periodontitis. The second-to-be-approved SDD is called Oracea, an effective systemic treatment for the chronic inflammatory skin disease, *rosacea*. Oracea (40 mg q.d.) was designed to produce low, non-antibacterial blood levels of doxycycline like Periostat (*<*1 µg/ml, typically about 0.5 µg/ml), in contrast to the much higher ‘peak’ blood levels, 2–5 µg/ml, produced by traditional antibiotic regimens of doxycycline (100 mg q.d. or b.i.d.). However, Oracea is a *NOVEL* customised formulation (capsule) combining 30 mg immediate-release doxycycline beads plus a unique 10 mg delayed/sustained-release version of the drug so that the patient only has to take the medication once/day rather than every 12 hours which improves compliance and favours greater long-term efficacy (20, 66).

In brief, rosacea patients typically exhibit erythema patches on facial skin together with (sometimes without) telangiectasia – swollen and permeable ‘spider-like’ veins on the nose and cheeks – as well as pustules and papules and other clinical manifestations, including ocular alterations (blepharitis). The success of SDD treatment of this disease has been attributed to its pleiotropic non-antibacterial actions (20, 66), including (but not limited to) MMP-inhibition, free radical scavenging, inhibition of inducible nitric oxide synthase and suppression of VEGF, and Monk et al. (20) described a series of multi-centre, double-blind, placebo-controlled clinical trials which demonstrated safety and efficacy in over 600 patients with rosacea using SDD (most of these studies used the sustained-release formulation).

Similar findings have been reported using both types of SDD formulations, and in a similar large number of patients, in multi-institutional clinical trials on efficacy in acne. Moreover, similar non-antibacterial (both anti-inflammatory and MMP-inhibitory) mechanisms have been invoked to explain the efficacy of these ‘low-dose’ doxycycline formulations in this most common of all inflammatory skin diseases (20, 66).

And, finally, the reader is referred to the review by Monk et al. (20) for a detailed discussion of the potential of various TCs to treat other dermatologic diseases by non-antibacterial mechanisms, such as granulomatous diseases (e.g. sarcoidosis), bullous and ulcerative disorders (e.g. bullous pemphigoid, epidermolysis bullosa, α_1_-antitrypsin-deficiency panniculitis), as well as non-healing wounds on the skin of diabetic patients (67, 68), and anti-angiogenesis on the skin (and other organs) of patients with Kaposi's sarcoma (20).

### Arthritis

Several clinical trials have explored the therapeutic potential of non-antibacterial properties of TCs in RA. These studies were recently reviewed by Greenwald (69), and earlier by Golub et al. (11, 14). Accordingly, a brief summary of these studies is now presented with a particular focus on a long-term placebo-controlled clinical trial, incorporating a subantimicrobial-dose regimen of doxycycline, carried out by O'Dell and his associates (70).

As reviewed by Greenwald (69), most of the clinical trials on TCs and RA tested minocycline, and ‘in general, mild beneficial effects were noted with respect to joint swelling and/or tenderness, laboratory parameters, patient assessment, etc’. Regarding therapeutic mechanisms, substantial inhibition of MMP (i.e. collagenase) activity, extracted from synovial tissues in patients treated with minocycline prior to total knee replacement surgery, was an exciting observation 25 years ago (71). Subsequent use of animal models, the adjuvant arthritic rat treated with TCs or non-antimicrobial CMTs, generated data consistent with these clinical findings in RA patients. Of particular interest, when the TCs or CMTs were orally administered to the arthritic rats in combination with an NSAID such as flurbiprofen (the latter, although it did, as expected, reduce signs of inflammation in the diseased joints, had no effect on collagenase activity), the anti-collagenase activity of the TCs was dramatically enhanced and the ‘radiologic bone damage’ was normalised (72). One mechanism identified in later studies was the ability of the NSAID to dramatically increase the level of the anti-collagenase TC delivered by the circulation to the inflamed joint (73). An additional clinical implication of this series of *in vivo* studies was the ability of the TCs and CMTs to reverse the loss of mechanical strength of the bones in these arthritic animals (72).

With this background in mind, O'Dell et al. (70) carried out the following clinical study on RA patients administered either antimicrobial-dose (100 mg, b.i.d.) or SDD (20 mg, b.i.d.) over a 2-year period. In brief, 66 patients with early RA (seropositive for rheumatoid factor) were randomly distributed into three experimental groups (note that the different experimental treatments, including those patients receiving placebo were all adjunctive to treatment with the potent anti-inflammatory drug, methotrexate). Essentially, the two groups of RA patients treated with either antimicrobial-dose doxycycline (ADD), or SDD, showed the same reduction in RA severity, assessed by American College of Rheumatology global scores, including decreases in the number of tender joints, swollen joints, joint pain, etc., and this benefit was 2–3 times greater than the improvement exhibited by the control group which received placebo capsules adjunctive to methotrexate. Moreover, the SDD group showed the same number of adverse events over the 2-year protocol as the placebo group, both of which were lower than those exhibited by the ADD group.

The therapeutic potential of the TCs in osteoarthritis (OA) has also been documented extensively particularly using animal models of this disease. One example was the ability of doxycycline, administered orally, to virtually ‘normalise’ the OA lesions in the dog model which involved transection of the anterior cruciate ligament of the knee (74). Subsequent studies implicated the ability of doxycycline to inhibit MMP (collagenase and gelatinase) activity in extracts of human osteoarthritic cartilage as mechanisms for their therapeutic potential in this disease (75). Although clinical trials on the use of TCs in OA are lacking (and difficult to design), one pilot study attempted to address this issue in oral disease (76). In three patients diagnosed with OA of the temporomandibular joints, based on clinical signs plus arthroscopy, a dramatic reduction of collagenase and gelatinase activities was seen in aspirated synovial fluids after a 3-month regimen of doxycycline, consistent with the studies by the Brandt group (74, 75).

Clearly, the experimental data on the efficacy of the RA and OA are intriguing and provide additional evidence in support of the non-antibacterial therapeutic properties of the TCs. However, their application as therapeutic agents in the management of arthritic diseases, including possible combinations with anti-inflammatory drugs, requires much more investigation, especially in human studies and clinical trials.

### Lymphangioleiomyomatosis

This is a rare and fatal lung disease affecting primarily women and characterised by uncontrolled infiltration of the lungs by smooth muscle cells (77). The current view on pathogenesis of this disease is that excessive production of MMPs by these smooth muscle cells slowly degrades the elastic fibres and collagen (the parenchyma) of the lungs, slowly and inexorably destroying lung function (78). As described by Folkman and his group (77), patients with this rare disease exhibit extremely high levels of MMP-2 and MMP-9 in their urine, and these biomarkers are considered diagnostic indicators of the severity of the disease. When Folkman and colleagues began treating a patient off-label with the only MMP inhibitor approved by the FDA for any disease, SDD (doxycycline, 20 mg b.i.d.), within weeks the urinary MMP levels decreased, followed by physiologic improvements in lung function (decreased PCO_2_ and increased PO_2_). Subsequently, the dose of doxycycline was increased to 100 mg per day and, ultimately, the patient's clinical response was so impressive she was ‘taken off the waiting list for a lung transplant – an outcome predicted by the decreasing urinary levels of MMPs’. As described by Folkman, other patients with similar lung-destructive disease also responded but further investigation is clearly required.

### Type II diabetes

To date, the only published clinical trial which assessed the efficacy of a non-antibiotic formulation of a TC on diabetic patients is the one recently described by Engebretsen and Hey-Hadavi (79). However, of relevance to their report, a series of earlier studies on type I and type II diabetic rats demonstrated that the non-antibiotic properties of TCs were effective in reducing the severity of a variety of abnormalities, including (but not limited to) those in collagen structure and turnover and in bone remodeling (both locally in the oral tissues and systemically), that contribute to the pathogenesis of diabetic complications, such as unusually severe periodontitis (see 11, 14, 15 and 80 for reviews). In brief, these early studies demonstrated that TCs, although they consistently did not reduce blood glucose levels, did produce the following beneficial effects in the severely hyperglycemic diabetic rat:

- TCs reduced pathologically excessive collagen degradation in gingiva and skin – this therapeutic effect reflected a ‘normalisation’ of excessive intracellular degradation of procollagen, as well as inhibition of MMP-mediated extracellular collagenolysis.
- TCs reduced the severity of both alveolar bone loss and systemic bone loss or osteoporosis.
- TCs improved wound healing in skin, an observation in diabetic rats that has been translated into clinical application. In this regard, several reports (67, 68) have described the clinical efficacy of topically applied doxycycline in non-healing wounds of diabetic patients, a benefit attributed to the inhibition of excessive MMP activity in wounds by this TC.
- TCs, including doxycycline and the chemically modified NON-antibacterial CMTs, particularly CMT-8 (chemically-modified doxycycline), reduced non-enzymatic glycation of proteins in serum and skin, reduced proteinuria, and prevented cataract formation (80).

With this background in mind, the study on diabetic patients administered a non-antibacterial formulation of doxycycline is now summarised with a primary focus on blood levels of glycosylated hemoglobin (HbA_1C_) (79). In brief, 45 type II diabetics were randomly assigned to either of the three experimental groups (*n =* 15 subjects/group), including those administered either placebo or an ADD (100 mg tablets) or SDD (20 mg). All subjects in the three groups were given sufficient tablets to be administered one tablet every 12 hours over a 3-month period, recognising that the ADD subjects only received drug for 2 weeks (and matching placebo tablets thereafter) while the placebo and SDD groups were administered their medications over the entire 3-month protocol; all subjects received SRP at the beginning of the study. The data indicated that although non-surgical periodontal therapy (SRP), with or without a 2-week regimen of antibiotics (ADD), did not produce a detectable improvement in HbA_1c_ levels (note that ‘HbA_1c_ is widely used as a surrogate measure for glycemic control and treatment decision making in clinical medicine’), the 3-month regimen of adjunctive SDD did produce statistically- and clinically significant evidence of improvement. Clearly, these preliminary observations, in this important area of great interest to medicine and dentistry, require confirmation and longer-term clinical trials.

### Non-antibacterial TCs and pathologic bone loss

#### *In vitro* and *in vivo* experimental models

Soon after the discovery that TCs can inhibit pathologically excessive MMP levels and activity (11, 14), the therapeutic potential of this non-antibiotic property of these drugs was explored for the treatment of pathologic bone loss. The rationale was clear: about 90% of the organic matrix of bone is composed of type I collagen (other constituents include various non-collagenous proteins, such as osteocalcin and bone sialoprotein, as well as proteoglycans and proteolipids) and the breakdown of this matrix during bone resorption, after dissolution of the hydroxyapatite mineral phase, is mediated by collagenases (MMP-1, MMP-8 and MMP-13) aided by additional MMPs (e.g. MMP-12 or macrophage metalloelastase which is also produced by osteoclasts) and other neutral- and- acid proteinases (81, 82). In this regard, several reports in 1984 described the ability of various TCs to inhibit osteoclast-mediated bone resorption in tissue culture induced either by parathyroid hormone or by bacterial lipopolysaccharide (endotoxin) (83, 84). Moreover, early *in vivo* studies demonstrated that TCs administered to rats with either experimentally induced type I diabetes or surgically induced menopause significantly reduced the severity of both local (periodontal) as well as systemic (osteoporosis) bone loss during these (and other, i.e. microbially induced alveolar bone loss) disease states (13, 14, 81). Although inhibition of excess collagenase activity by TCs, particularly a potent non-antimicrobial formulation of doxycycline (CMT-8), as well as reduced bone resorption, played a role in preventing local and systemic bone loss (11, 13, 14, 81), beneficial effects on osteoblast activity and on bone formation were also observed. As examples, suppressed osteoblast activity and reduced bone formation rates during diabetes were increased *in vivo* by oral administration of non-antimicrobial TCs (82, 85); evidence suggests that an increase in steady-state levels of type I procollagen mRNA and accelerated collagen synthesis provides one mechanism by which TCs counteract the loss of collagen (or atrophy) which occurs as a complication of diabetes in bone and other tissues such as skin (86). Also, in the animal model of PM osteoporosis, the ovariectomised aged rat, TCs were found to substantially increase the rate of bone formation, as well as inhibit bone resorption, as mechanisms which overcome the estrogen-deficiency-induced loss of bone density (87, 88).

With this background in mind, Payne et al. (89) over a decade ago carried out a preliminary double-blind, placebo-controlled study on PM women diagnosed with both periodontal/alveolar bone loss and systemic bone loss/osteoporosis. In brief, these subjects were administered, over a 1-year period, a ‘cyclical’ regimen (4 months ‘on’ drug, 4 ‘off’ drug and 4 months back ‘on’ drug) (90) of either placebo or SDD capsules adjunctive to routine periodontal debridement (periodontal maintenance therapy), the latter to reduce the bacterial ‘burden’ in periodontal pockets. Of extreme interest, the SDD regimen appeared to reduce the *progressive* loss of both alveolar bone density and alveolar bone height, and reduced progressive loss of soft tissue support. In summary, the above-described basic laboratory experiments and preliminary clinical studies provided the rationale for the extensive NIH-supported, two-institution clinical trial discussed below.

#### Clinical research on post-menopausal women treated with SDD

The details of this recent clinical trial, including the extensive clinical, radiologic, biomarker and statistical analyses, as well as safety data, have been described in a series of publications which were recently reviewed by Payne and Golub (56). To summarise, the objective of this placebo-controlled, double-blind randomised clinical trial (funded for 7 years by the NIH), carried out at two university centres, University of Nebraska and Stony Brook University, was to determine whether *long-term* administration of SDD to PM women, who exhibited *both* local bone loss (periodontitis) as well as mild systemic bone loss (osteopenia), would be safe and beneficial for *both* conditions. After screening 675 subjects, using extensive inclusion and exclusion criteria (56), 128 subjects were equally randomised to either of the two treatments, a daily regimen of SDD or placebo every 12 hours over a 2-year period; note that all subjects received periodontal maintenance therapy (mechanical debridement) and calcium and vitamin D supplements during the trial.

In brief, the 2-year regimen of SDD adjunctive to periodontal maintenance therapy produced the following periodontal benefits (and systemic benefits described later) consistent with the earlier pilot study (89) already described:

- Significantly reduced progressive loss of periodontal attachment based on intent-to-treat analysis (ITT), and bleeding on probing in subgroups.
- Significantly reduced the progressive loss of alveolar bone density in pocket sites *exhibiting* periodontitis (i.e. pockets characterised by ‘baseline probing depths 5 mm or greater’).
- Significantly reduced biochemical markers of periodontal disease severity in GCF, including collagenase activity, particularly that derived from acute inflammatory cells, MMP-8 (ITT), and the bone resorption diagnostic marker ICTP (a telopeptide pyridinoline-crosslink degradation fragment of bone type I collagen monitored in medical diagnostics); moreover, in a subgroup of these women, SDD also reduced IL-1β in periodontal pockets.

Also, of extreme interest were the serum analyses and safety data which reflected the subject's medical/systemic response to long-term SDD. In this regard, the diagnostic biomarkers of bone formation in the serum samples from these patients, bone-specific alkaline phosphatase and osteocalcin, were not affected by the 2-year regimen of SDD treatment. However, the bone resorption biomarker, ICTP (which was reduced in the periodontal pockets; see above), as well as a second and newer biomarker of bone destruction, CTX (deoxypyridinoline-crosslink fragment of type I collagen), both were reduced in the serum of subgroups of these PM women; e.g. those subjects who exhibited high-performance liquid chromatography-detectable doxycycline (median=0.59 µg/ml) in their serum. As a result, we recently proposed that SDD could reduce the risk of conversion of mild systemic bone loss (osteopenia) into the more severe form of bone deficiency disease, osteoporosis, the latter (but not the former) requiring medical intervention (91).

And finally, the PM subjects treated with long-term SDD exhibited no Adverse Events (AEs) significantly different from placebo (including no microbially related events) except for a dramatic and statistically significant reduction in dermatologic AEs (e.g. acne, rosacea and rash) compared to those women administered placebo tablets; note, these findings were consistent with the clinical studies on chronic inflammatory skin diseases described earlier (20).

### Cardiovascular disease

As reviewed recently (23, 92), TCs by non-antimicrobial mechanisms have demonstrated therapeutic potential in several cardiovascular diseases (CVDs), including (a) reduced severity of hypertension by inhibiting MMP activity which prevents the degradation of elastic and collagen fibres in addition to reversing the abnormal remodelling of blood vessel walls (the ability of doxycycline and the CMTs to inhibit MMP-9 and MMP-12 also reduces the severity of experimentally induced and *clinical* aortic aneurysms [93, 94]) and (b) reduced severity of atherosclerosis which reduces the risk for acute myocardial infarction (AMI). As discussed below, SDD has been found in clinical trials to reduce biomarkers in the circulation which mediate atherosclerosis, including MMP-8 and MMP-9, as well as markers of systemic inflammation, C-reactive protein and IL-6.

These therapeutic responses to SDD administration could be responsible for preventing the breakdown of the collagen ‘cap’ coating the cholesterol-rich atheroscleromatous plaques lining coronary (and other) arteries, which decreases the risk of plaque rupture, thrombosis and AMI. Additional mechanisms by which doxycycline and other TCs could provide cardioprotection have been identified by Schulz and his colleagues (95). In brief, they observed that doxycycline penetrates subcellular organelles within cardiac myocytes and inhibits MMP-2 mediated degradation of intracellular contractile proteins, including alpha-actinin, myosin light chain-1 and troponin-I.

Regarding clinical trials which addressed the efficacy of TCs in CVDs, the rationale for this application was greatly enhanced by a major case-control study reported by Meier et al. in 1999 (96). As summarised by us previously (92), Meier's group analysed data from 3,315 cases ‘with a diagnosis of first-time acute myocardial infarction’, plus 13,139 control patients who did not experience CVD, and recorded their use of various types of antibiotics over a 5-year period. In brief, those patients who were treated for infection with TCs showed a statistically significant reduction in the incidence of AMI, whereas those on other antibiotics such as ‘macrolides (primarily erythromycin), sulfonamides, penicillins, or cephalosporins’ showed no effect – initial examination of the quinolone antibiotic data suggested a borderline therapeutic effect which was not supported by subsequent statistical analysis (97). Meier et al. (96) suggested that the likely explanation was the suppression by TC of *Chlamydia pneumoniae*, a microorganism thought, at that time, to play a role in CVD. However, we proposed (98) that TCs were effective in reducing the incidence of AMI, and the other antibiotics were not, because only the TCs exhibited the unique non-antimicrobial mechanisms (especially MMP-inhibition, but also the ability to suppress the inflammatory mediators, including cytokines, prostanoids, nitric oxide and the reactive oxygen species) discussed above which would protect the vulnerable atheroscleromatous plaques, lining the coronary and other arteries, from rupture and subsequent cardiac events.

Recent long-term multi-institutional clinical trials have not shown any evidence of efficacy of antimicrobial therapy (e.g. azithromycin) in patients with CVD (99, 100). In contrast, several recent double-blind, placebo-controlled studies demonstrated that SDD reduced biomarkers of systemic inflammation strongly associated with CVD (101–103), including the tissue-destructive proteinase, MMP-9, the long-term proinflammatory cytokine, IL-6, and the acute-phase protein, C-reactive protein. In addition, this non-antibiotic formulation of doxycycline also increased the cardio-protective HDL cholesterol in the circulation of patients (at least in subsets of these subjects) with a history of (102) or at risk for CVD (103). These reports are also consistent with the ‘Jupiter’ study (104) which demonstrated that a ‘statin’ drug (rosuvastatin) administered to large numbers of subjects ‘without hyperlipidemia’ reduced cardio-vascular events associated with the ability of this drug to reduce the levels of high-sensitivity CRP in the circulation.

## Conclusion

The major hurdle to more wide-spread clinical implementation that this novel (and government-approved) SDD-host-modulation-therapy has had to overcome is the long history of TCs usage *only* as antimicrobial agents for treatment of infection. However, the vast amount of data generated by numerous dental and medical clinical trials continues to support the use of SDD in the treatment of chronic inflammatory diseases. Thus, the view, first expressed more than two decades ago (14), that non-antimicrobial TCs (the SDD formulations and, in the future, CMTs) are not only safe and effective in the management of periodontal disease but would also be therapeutically useful in a variety of medical disorders, seems much closer to realisation at this time.
